# Ionomics and proteomics analysis of the pancreatic repair in a murine severe acute pancreatitis model treated with Qingyi decoction

**DOI:** 10.3389/fimmu.2026.1797100

**Published:** 2026-04-22

**Authors:** Minla Rao, Suzhen Zhang, Wenbin Lai, Di Zou, Zhihuang Wu, Zhenggang Yin, Wendie Yu, Weihua Zheng, Huimin Li, Yong Zhou, Wenjin Fu, Shayan Chen

**Affiliations:** 1 Department of Laboratory Science, Binhaiwan Central Hospital of Dongguan, Dongguan, Guangdong, China; 2 Dongguan Key Laboratory of Accurate Etiological Research on the Pathogenesis of Inflammation and Cancer, Dongguan Science and Technology Bureau, Dongguan, Guangdong, China; 3 Department of Laboratory Science, Dongguan Dongcheng Hospital, Dongguan, Guangdong, China; 4 Department of Laboratory Science, Dongguan Hospital of Integrated Traditional Chinese and Western Medicine, Dongguan, Guangdong, China; 5 Department of Laboratory, Houjie Hospital and Clinical College of Guangdong Medical University, Dongguan, Guangdong, China; 6 Central Laboratory, Binhaiwan Central Hospital of Dongguan, Dongguan, Guangdong, China

**Keywords:** ionomics, oxidative phosphorylation, proteomics, Qingyi decoction, severe acute pancreatitis, zinc

## Abstract

**Background:**

Current therapeutic options for severe acute pancreatitis (SAP) remain limited. The integration of traditional Chinese and Western medicines offers a promising approach for patients with SAP, with Qingyi decoction (QYD) emerging as a classic and effective formula for this condition. Nevertheless, its precise mechanism—particularly regarding ionomics and proteomics—requires further elucidation.

**Purpose:**

This study aimed to investigate the possible underlying mechanisms by exploring the characteristics and relationships of alterations in ionomics and proteomics profiles during pancreatic repair in a mouse model of SAP treated with QYD.

**Methods:**

The therapeutic efficacy of QYD in SAP was validated through pathological section analysis and immunohistochemistry. Total elemental analysis of serum and mouse tissues was performed using inductively coupled plasma–mass spectrometry to identify relevant target elements. XPS analysis was employed to determine the chemical speciation of the target elements. Proteomic analysis, combined with KEGG, GO enrichment and STRING protein–protein interaction analyses, elucidated the protein molecules and biological pathways and the core proteins involved in QYD-mediated SAP treatment were verified by Western blotting. Finally, to clarify the relationship between changes in ion content and alterations in oxidative phosphorylation-related proteins and mitochondria-associated proteins, we conducted Pearson correlation analysis and mitochondrial morphological identification.

**Results:**

QYD effectively alleviated symptoms of lipopolysaccharide-induced SAP. During this process, metal ions—particularly zinc ions—underwent significant coordination changes. Five core proteins were extracted from the proteomics section, and oxidative phosphorylation emerged as a critical pathway in this therapeutic mechanism. Furthermore, correlation analysis revealed close connections between ionic changes (Zn, Cu, Fe, Ca, Mg, Mn, and Se) and the expression profiles of oxidative phosphorylation-related proteins (Nduf and Atp5f1b) and mitochondrial-associated proteins (Tfam, Ppp3ca, and Myef2), which were also consistent with the observed changes in mitochondrial morphology.

**Conclusion:**

The therapeutic mechanism of QYD in SAP may involve the ionic spectrum, especially zinc, modulating disease progression through mitochondrial oxidative phosphorylation.

## 1 Introduction

Acute pancreatitis is a common gastrointestinal disorder whose incidence has increased globally in recent decades (1). Approximately 10–20% of patients develop SAP, a life-threatening condition characterized by high mortality rates, acinar cell necrosis, oxidative stress, and mitochondrial dysfunction (2, 3). Despite extensive research, the molecular mechanisms underlying SAP pathogenesis remain incompletely understood (4), and effective targeted therapies are still lacking.

Emerging evidence has highlighted the critical role of metal ions in SAP pathophysiology (5). For example, zinc is an essential cofactor for numerous enzymes involved in antioxidant defence (e.g., Cu/Zn-superoxide dismutase) and mitochondrial function (6). Our previous research revealed that metalloproteins exhibit characteristic alterations in acute pancreatitis (7). However, the landscape of metal dysregulation in SAP—beyond individual metals—remains largely unexplored.

QYD, a representative prescription of Tong Li Gong Xia Fa, is a traditional Chinese medicine formula composed of twelve herbal components and has been clinically used to treat SAP in China for decades (8). Previous studies have suggested that QYD may have anti-inflammatory and antioxidative effects (9); however, the molecular mechanisms—particularly whether it modulates metal homeostasis and thereby influences protein expression—have not been systematically investigated (10–12).

Given the potential link between metal dysregulation and key pathological processes in SAP, we hypothesized that QYD may exert therapeutic effects by restoring metal homeostasis and consequently modulating metal-dependent biological pathways. To test this hypothesis, we employed an integrated ionomics and proteomics approach. Ionomics is an emerging, cutting-edge, comprehensive and interdisciplinary field that has gradually developed following genomics, proteomics and metabolomics (13, 14). It investigates the distribution, content, chemical forms, structures and functions of all metallic atoms within living organisms, whether free or complexed (15). This approach enables comprehensive profiling of metal elements, including their concentrations and chemical speciation, in biological samples (16), providing an unbiased view of metal dysregulation. Proteomics allows global analysis of protein expression changes (17), revealing downstream functional pathways affected by metal modulation. To date, no published studies have combined ionomics and proteomics in SAP. The integration of these two omics approaches offers a unique opportunity to link metal changes to functional outcomes at the systems level. Therefore, research from the perspective of “metal proteins” may elucidate the complex mechanisms of SAP.

In this study, we aimed to (1) characterize the alterations in pancreatic metal ions in SAP and their responses to QYD treatment; (2) identify differentially expressed proteins and pathways modulated by QYD; and (3) integrate ionomics and proteomics data to elucidate the potential links between metal homeostasis and functional pathways.

## 2 Materials and methods

### 2.1 Animals and treatment

#### 2.1.1 Mice

Mice of the C57BL/6J strain, aged 6–8 weeks and weighing 17–23 grams, were procured from Beijing Vital River Laboratory Animal Technology Co., Ltd. (licence number: SCXK 2018–0006). They were kept in an SPF-certified, pathogen-free environment. All animal experiments in this study (NO: 20180202-0005) received approval from the Animal Care and Use Committee at the involved institutions (Tianjin Hospital of Traditional Chinese Medicine).

#### 2.1.2 SAP animal model

The animals were assigned to experimental groups using a block randomization method. Details about the number of animals, replicates, and experimental runs can be found in the figure legends, and these were selected based on the standard deviation (S.D.). The mice were randomly divided into three cohorts: (1) negative control (CON) (n=10), (2) SAP model (SAP) (n=10), and (3) QYD treatment in the SAP model (QYD+SAP) (n=30) There are three doses: high, medium, and low (5g/kg, 10g/kg, 20g/kg), with 10 mice per dose. The establishment of the SAP model was described previously. Briefly, following a 12-hour fasting period with unlimited access to water, the mice in the SAP and QYD+SAP groups were treated. The CON group was not subjected to any treatment. The SAP and QYD+SAP groups of mice received subcutaneous injections of 100 μg/kg caerulein once per hour for seven consecutive doses and 10 mg/kg lipopolysaccharides (LPS) once per hour for three consecutive doses.

For QYD treatment, mice in the group treated with QYD were given oral doses of QYD at 10 g/kg, three times the usual clinical concentration. One was immediately after SAP induction (0 hours), and the other was after 12 hours; the third was after 24 hours. Following QYD treatment, serum and pancreatic samples were obtained from the mice, and tissue was fixed with 4% paraformaldehyde for further analysis, including IHC and HE staining for pathological sections.

### 2.2 Reagents

QYD is a Chinese herbal medicine formulation whose primary ingredients include *Rheum officinale Baill* (Dahuang, *Rhei Radix et Rhizoma*), *Artemisiacapillaris Thunb* (Yincheng, *Artemisiae Scopariae Herba*.), *Gardenia jasminoides Ellis* (Zhizi, *Gardeniae Fructus*), *Forsythiae Fructus* (Lianqiao, *Forsythiae Fructus*), *Mirabilite* (Mangxiao, *Natrii Sulfas*), *Lonicera japonica* (Jinyinhua, *Lonicerae Japonicae Flos*), *Angelica sinensis* (Danggui, *Angelicae Sinensis Radix*), *Corydalis Rhizoma* (Yanhusuo, *Corydalis acropteryx Fedde*), *Zingiber officinale costus* (Muxiang, *Aucklandiae Radix*), *Glycyrrhiza uralensis Fisch, ex DC* (Gancao, *Glycyrrhizae Radix et Rhizoma*), *Bupleurum chinense* (Chaihu, *Bupleuri Radix*), and *Paeonia lactiflora Pall* (Baishao, *Paeoniae Radix Alba*). The botanical nomenclature was verified using The Plant List database (https://powo.science.kew.org/) and Medicinal Plant Names Services (http://mpns.kew.org).

All raw herbs for the formula, except *Natrii Sulfas*, were soaked and boiled in water that was ten times their mass (1710 ml) for one hour, followed by filtration through six layers of gauze. Next, 20 grams of *Natrii Sulfas* was added to the extract and boiled for an additional 30 minutes. The filtrates were combined and vacuum-evaporated to reach a final volume of 191 ml (equivalent to 1 g crude herb/ml) after being filtered with six layers of absorbent gauze when the solution was hot. The extract, which had a yield of 21.2%, was stored in a desiccator until needed.

The medicinal herbs were compared with the Pharmacopoeia of the People’s Republic of China (2015 edition; Chinese Medical Science and Technology Press) and procured from Anguo Longlian Prepared Chinese Herbal Medicine Co., Ltd. (Hebei Province, China). The products met the commercial quality standards specified in the Chinese Pharmacopoeia (2010).

**Figure 1:**
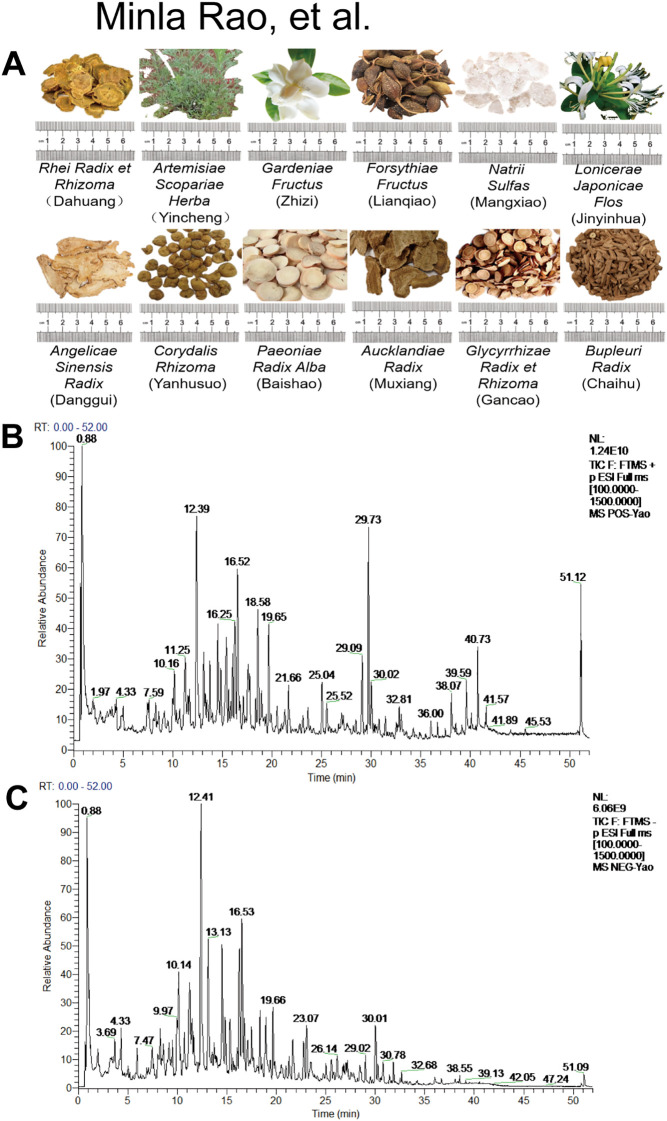
Qualitative analysis of QYD. **(A)** Composition of the drugs in QYD. **(B)** In positive ion mode, the total ion chromatogram of QYD. **(C)** The total ion chromatogram for QYD when using negative ion mode.

We also conducted UHPLC–QTOF–MS analysis on QYD. Specifically, 1.0 g of freeze-dried QYD powder was subjected to ultrasound-assisted extraction for 30 minutes in 50 ml of methanol/water (1:1, v/v). The solution was centrifuged at 13,000 rpm for 10 minutes at 4 °C, and the supernatant was filtered through a 0.22 μm membrane before 1.0 μl was injected into the UHPLC–QTOF–MS for analysis; the results are shown in **Figures 1AC**.

**Figure 2:**
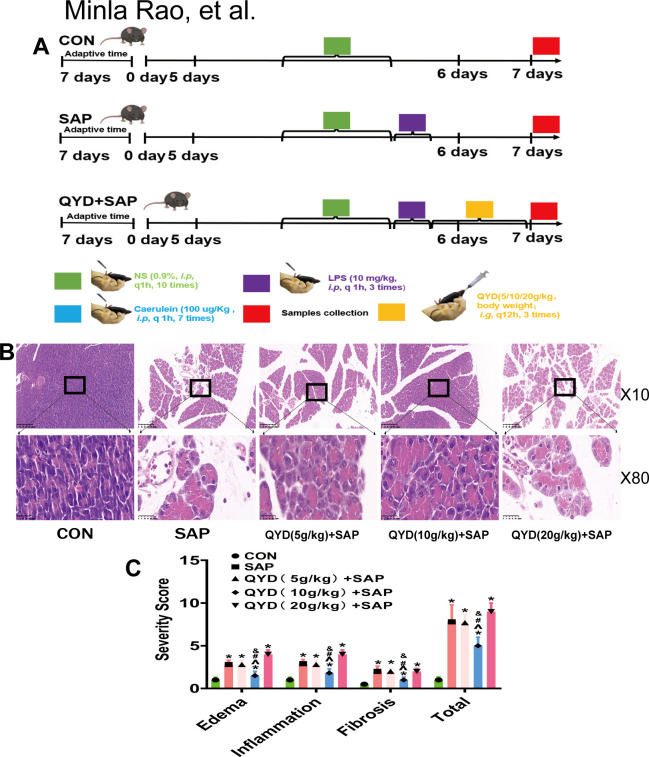
Establishment and validation of the mouse models. **(A)** Treatment scheme of each group (n=10 mice per group). **(B)** Histological examination (H&E staining) of pancreatic sections from the established QYD treatment models at varying concentrations. Scale bars: 200 µm for 10× and 25 µm for 80× views. **(C)** Quantification of edema, inflammation, and acute collagen deposition-induced fibrosis in mice using different concentrations of QYD. 6 randomly selected fields per mouse. The number for each experiment was 10 per group. Data represent mean ± *SD*; Statistical significance was determined by one-way ANOVA followed by Tukey’s *post hoc* test; (*, vs CON, *p* < 0.05; ^ vs SAP, *p* < 0.05; #, vs 5g/kg QYD+SAP, *p* < 0.05; &, vs 20g/kg QYD+SAP, *p* < 0.05).

In the experiments, granules containing the 12 ingredients were dissolved in an appropriate volume of ddH_2_O, and the resulting solution was refrigerated and packaged at a concentration of 1 kg/L. To determine the optimal dosage of QYD we evaluated three dose levels (5, 10, and 20 g/kg) based on the references (9, 18). The 10 g/kg dose demonstrated superior efficacy (**Figures 2B, C**) and was selected for subsequent administration via oral gavage, in accordance with validated protocols. Moreover, this dosage, derived from the human equivalent dose based on body surface area, falls within a clinically relevant range while minimizing artefacts associated with gastric distension and ensuring dosing consistency.

Caerulein and LPS were purchased from Macklin and Sigma–Aldrich, respectively. Stock solutions were prepared in ddH_2_O at concentrations of 1 mg/mL (caerulein) and 2 mg/mL (LPS) and diluted to working concentrations as needed.

### 2.3 Haematoxylin–eosin staining, IHC and ELISA

Pancreatic tissue samples fixed in 4% paraformaldehyde were embedded in paraffin and sectioned at a thickness of 5 μm. Sections were stained with HE for histological evaluation. Pathological changes—including oedema, inflammation, acinar cell necrosis, and fibrosis (acute collagen deposition)—were assessed by two independent pathologists who were blinded to group allocations.

For IHC detection of amylase (AMY) and macrophages (F4/80^+^CD11b^+^), the sections were deparaffinized, rehydrated, and subjected to antigen retrieval. After blocking with 5% bovine serum albumin, the sections were incubated with anti-AMY and anti-F4/80 antibodies overnight at 4 °C, and primary antibody working solution was added dropwise. For IHC double-staining, sequential labelling was used to avoid cross-reactivity. After the cells were washed with PBS the next day, they were incubated with a secondary anti-CD11b antibody at room temperature for one hour, followed by an HRP-conjugated secondary antibody. Diaminobenzidine (DAB) was used as the chromogen, and the sections were counterstained with haematoxylin. Images were captured using a light microscope (Olympus, Japan), and positive cells were quantified using ImageJ software (NIH, USA). For quantitative analysis, five nonoverlapping high-power fields (HPFs, 64× and 40× magnification) were randomly selected per section. The integrated optical density (AMY) was measured using ImageJ software (v1.50i), and positive cells (macrophages) were counted manually with consistent threshold settings applied across all samples. The average value from the two investigators was used for statistical analysis (19).

Serum levels of AMY activity, C-reactive protein (CRP), myeloperoxidase (MPO), monocyte chemoattractant protein-1 (MCP-1), and lactate dehydrogenase (LDH) were measured using commercial ELISA kits according to the manufacturers’ protocols.

### 2.4 Metallomic analysis by ICP–MS

One hundred fifty microlitres of serum or 100 mg of pancreatic tissue sample was removed into digestion tubes, which were soaked in 30% HNO_3_ for at least 24 h, followed by another 24 h in high-purity water, and then rinsed again with high-purity water. Three mL of 65% HNO_3_ was added to the tubes, and these tubes were then placed into an airing chamber for 12 h. The samples were subjected to microwave digestion at different temperatures. The solutions were subsequently transferred into 50 mL centrifuge tubes, and the tubes were then washed with high ultrapure water three times to ensure that the HNO_3_ concentration was less than 5%. All these solutions were stored at 4 °C until analysis by ICP–MS.

Environmental calibration standard solutions of 10 mg/L of aluminium (Al), arsenic (As), barium (Ba), beryllium (Be), cobalt (Co), chromium (Cr), copper (Cu), manganese (Mn), nickel (Ni), lead (Pb), selenium (Se), vanadium (V) and zinc (Zn), in addition to 1000 mg/L of calcium (Ca), iron (Fe), potassium (K), magnesium (Mg), and sodium (Na), were diluted into a series of concentrations with 5% HNO_3_. Before ICP–MS analysis, the samples were purged with helium (99.99%) at 2 L/min. When the forward power reached 1500 W and the reflected power reached 1 W, it indicated that the system could start up. Furthermore, a calibration curve, the lower limit of quantification (LLOQ), a precision test and repeated experiments were carried out to validate the reliability of the method.

### 2.5 X-ray photoelectron spectroscopy

The sample was placed onto the sample stage. Following evacuation, a full-spectrum survey scan was performed using a Thermos Scientific K-Alpha instrument. A broad energy range (typically 0–1200 eV) was used to identify all the elements present on the sample surface (excluding H and He, whose photoelectron energies were extremely low and difficult to detect), and a preliminary assessment of their relative abundances was performed. A high-resolution scan was subsequently performed. This approach targets specific elements detected in the survey scan, employing a narrow energy range to obtain detailed spectral peaks.

### 2.6 Atomic absorption spectroscopy

Zinc concentrations in serum and tissue homogenate supernatants were determined using a graphite furnace atomic absorption spectrometer (PerkinElmer PinAAcle 900T, USA). Diluted samples were aspirated into an acetylene flame, and Zn concentrations were calculated automatically using fast sequential analysis with the associated Spectra software. Standard curves were generated using certified zinc reference solutions.

### 2.7 Proteomic analysis

Protein extraction and digestion, high-pH reverse-phase fractionation, LC–MS/MS analysis, and data processing and analysis were performed. Data-independent acquisition (DIA) of the samples was performed using an Astral Zoom mass spectrometer. Given the high resolution and rapid scanning rate of the instrument, sample acquisition was completed within 2 hours with minimal instrument-induced run-to-run variation. Thus, the spiking of heavy isotope-labelled standard peptides for instrument monitoring was omitted. The instrument status was routinely evaluated using standard peptides, and the HeLa peptide digest used for quality control strictly met the criteria for sample injection. The threshold criteria for identifying differentially expressed proteins (DEPs) were set as a fold change (FC) ≥ 1.2 or ≤ 0.83 (i.e., |log2FC| ≥ 0.263) combined with a p value < 0.05. Differential protein expression analysis was based on the relative quantitative values of the proteins.

### 2.8 Western blot analysis

Total protein was extracted using a radioimmunoprecipitation assay (RIPA), and protein concentrations were measured with a BCA protein assay kit. Equal quantities of protein were separated using 10% SDS–polyacrylamide gel electrophoresis and then transferred onto a polyvinylidene fluoride (PVDF) membrane (Millipore, USA). Afterwards, the membrane was treated with 5% nonfat milk in Tris-buffered saline (TBS). For quality control, primary antibodies against Exoc6b, Itih4, Klk1b16, GPX5, Il1r2, Samd9l, Mrpl4, Nup210, MPP2 from Affinity, and GAPDH from Abcam, Inc., in the USA were used. Proteins were detected using an enhanced chemiluminescence kit from Millipore (USA), and their expression levels were standardized to those of GAPDH for all the experimental groups; the value of the ratio of MPP2 to GAPDH was defined as 1.

### 2.9 Bioinformatics analysis

Bioinformatics analysis was performed to assess the functions, pathways, or interaction networks of the DEPs identified in pancreatic tissue. In terms of the significance of the results of the GO enrichment analysis for the DEPs using the DAVID database (https://davidbioinformatics.nih.gov/), the results are expressed as bar charts, with three types of bars representing the important gene functional categories. The results of the KEGG pathway enrichment analysis for the DEPs are displayed in bubble chart form, which presents the important up- and downregulated metabolic pathways. A PPI network of the differential metal proteins was constructed using data from the STRING database (https://string-db.org). The correlations between metal content and DEPs were classified, and gene enrichment analyses were performed (Hallmarks, GO, and KEGG). Cystoscope 3.9.1 was used for visualization, and the Clue GO plugin was used to extract hub proteins from the PPI network.

### 2.10 Scanning electron microscopy

Samples were fixed with 2.5% glutaraldehyde in 0.1 M phosphate buffer (pH 7.2) for 4 h. After postfixation with 1% OsO_4_, the samples were dehydrated in ethanol and embedded in Epon resin. Ultrathin sections (70 nm) were stained with uranyl acetate and lead citrate. Images were captured with a JEOL JEM-1400Flash transmission electron microscope operated at 80 kV.

### 2.11 Correlation analysis

To investigate the relationships between the ion spectrum and DEPs, a correlation analysis was performed between the proteins involved in oxidative phosphorylation or mitochondrial energy metabolism. The Pearson or Spearman correlation coefficient was calculated for each pairwise combination of ion concentration and gene expression level. The resulting correlation matrix was visualized using a heatmap, where rows typically represent differential genes and columns represent ion species (or vice versa). Within the heatmap, the colour intensity and hue reflect the magnitude and direction (positive or negative) of the correlation coefficient. Hierarchical clustering was often applied to both dimensions to group genes and ions with similar correlation patterns, thereby revealing underlying biological structures.

### 2.12 Statistical analysis

For ICP–MS analysis, all the results are presented as the mean ± *SD* and were analysed using SPSS 17.0 (SPSS, IBM, United States). Multiple comparisons for two groups were performed using independent sample t tests. A *p* value less than 0.05 was considered to indicate statistical significance.

For comparisons involving biological replicates of proteomics analysis, Student’s t test was employed, whereas the significance A/B test was applied for comparisons between single samples (without replicates) to calculate the corresponding *p* values. For multigroup comparisons with biological replicates, ANOVA was used. Conversely, for multigroup comparisons lacking biological replicates, the chi-square test or Fisher’s exact test was performed to calculate *p* values. A *p* value < 0.05 was considered the threshold for statistical significance to define DEPs across multiple groups.

The other results are reported as the mean ± *SD*. Statistical analysis was conducted using the SPSS 17.0 software package (SPSS, Chicago, IL, USA) and GraphPad Prism (GraphPad Software Inc., La Jolla, CA, USA). Comparisons between two groups were performed using an unpaired Student’s t test. Comparisons among multiple groups were performed using one-way ANOVA followed by Tukey’s *post hoc* test. Statistical significance was set at *p* < 0.05.

## 3 Results

### 3.1 Qualitative analysis of QYD with UPLC/MS-MS

The composition of the drugs in QYD (**Figure 1A**) were determined, and a UPLC/MS-MS analysis (**Figures 1B, C**) was performed. The specific major components of QYD are listed in **Supplementary Appendix 1**.

### 3.2 Establishment and validation of the SAP and QYD-treated SAP mouse models

A schematic diagram of the establishment of the mouse SAP and SAP+QYD models is shown in **Figure 2A**. The experimental design for different concentrations of QYD was as described above. The pancreas is a critical organ involved in the development of SAP in mice. As evidenced by microscopic analyses and histological assessments (**Figure 2B**) of the pancreatic tissue, the CON group demonstrated intact pancreatic architecture devoid of inflammation or necrosis. Conversely, the SAP group displayed morphological swelling, alveolar atrophy, acute collagen fibre deposition (an indicator of early tissue remodelling) and pancreatic tissue thinning. Notably, normal pancreatic structure was preserved in the QYD+SAP group, revealing that QYD treatment attenuated oedema, inflammation, and acute collagen fibres in the pancreatic tissue of SAP mice (**Figure 2C**). Our research indicated that the optimal concentration of QYD was 10 g/kg; therefore, all subsequent experiments employed this concentration. These results confirmed the successful establishment of SAP and the protective effect of QYD.

### 3.3 QYD alleviates the inflammatory response in SAP

**Figure 3:**
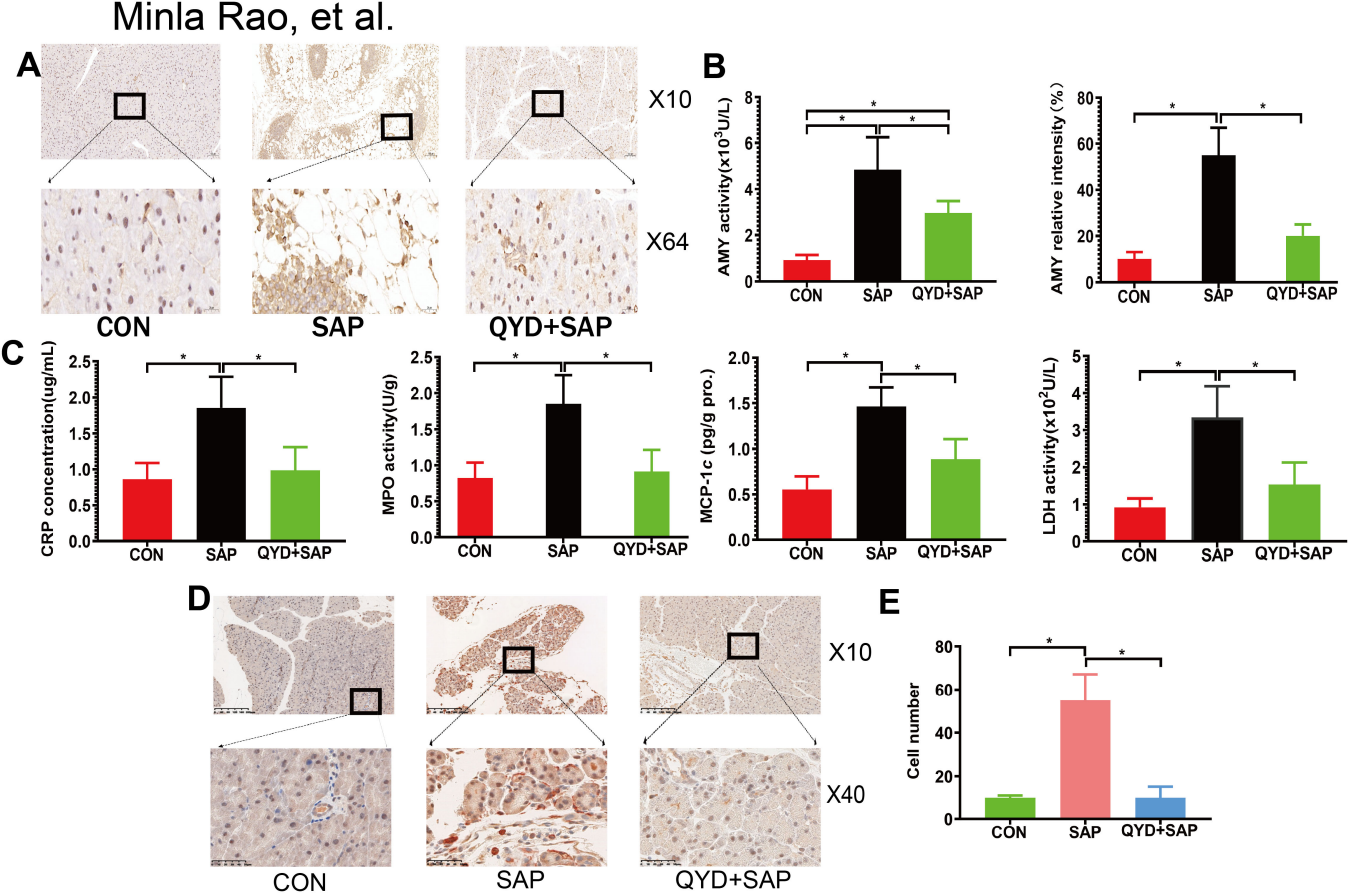
QYD alleviates the inflammatory response in SAP. **(A)** Immunohistochemical analysis of AMY expression in pancreatic tissues. Scale bars: 100 µm for 10× and 20 µm for 100× views. **(B)** Changes of activity in serum AMY of mouse in each group (left). Changes of AMY relative intensity in mouse (right). 6 randomly selected fields per mouse. **(C)** Changes in serum CRP, MPO, MCP-1c, and LDH levels of mice. **(D)** Immunohistochemistry showed that macrophages were stained with F4/80 as brownish yellow, CD11b as red, and the nucleus as blue. **(E)** The number of inflammatory cells. Data were presented as mean ± *SD*; n=10 independent biological replicates; Statistical significance was determined by one-way ANOVA followed by Tukey’s *post hoc* test. (*P < 0.05).

AMY is a digestive enzyme secreted by the pancreas. When the pancreas becomes inflamed, AMY is abnormally present in large quantities in both tissue and serum. Compared with those in the CON group, the intensity and activity of AMY in the SAP group were greater (**Figures 3A, B**), and the levels of CRP, MPO, MCP-1 and LDH (**Figure 3C**) and the number of inflammatory cells (macrophages) were significantly greater (**Figures 3D, E**). However, QYD treatment effectively suppressed the SAP-induced inflammatory response. Specifically, the findings demonstrated that QYD can inhibit the abnormal activation and leaky secretion of AMY in terms of pancreatic function. Moreover, in terms of the pancreatic microenvironment, the levels of CRP, MPO, MCP-1 and LDH were distinctly predictive and served as independent factors for predicting SAP. At the level of pancreatic cell tissue, the inflammatory infiltration of macrophages was decreased in the QYD treatment group. Collectively, these results demonstrated that QYD may have anti-inflammatory and protective effects.

### 3.4 Elemental profiling in mouse serum by ICP–MS

**Figure 4:**
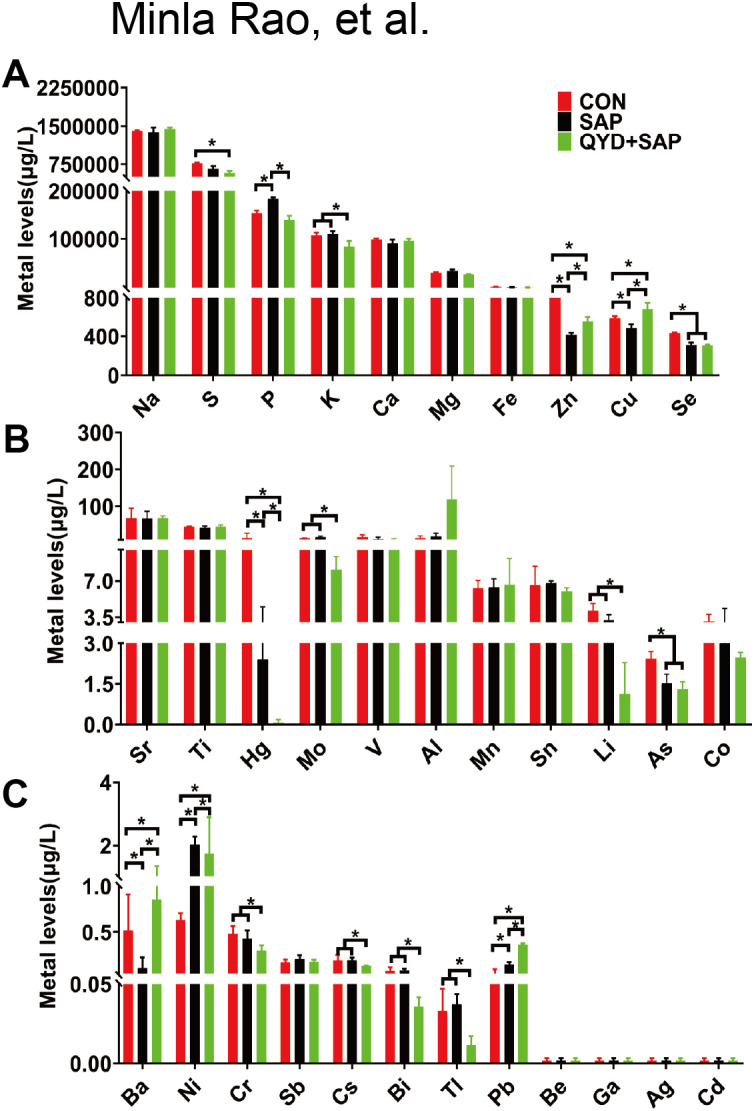
Elemental profiling in mouse serum by ICP–MS. **(A)** Comparative analyses of ion concentrations in plasma were conducted at high levels. **(B)** Comparative analyses of ion concentrations in plasma were conducted at medium levels. **(C)** Comparative analyses of ion concentrations in plasma were conducted at low levels. Data represent mean ± *SD*; n=3 independent biological replicates; Statistical significance was determined by one-way ANOVA followed by Tukey’s *post hoc* test. (*P < 0.05).

To investigate the characteristics of ionomics in pancreatic repair in a SAP model treated with QYD, we employed ICP–MS to analyse various metal elements present in serum. In mouse serum, we identified several elements that exhibited significant differences: S, P, K, Zn, Cu, Se, Hg, Mo, Li, and As (**Figures 4A–C**). The concentrations of the first six elements were higher, whereas those of the latter four were lower. Within living organisms, these elements may serve as enzyme cofactors, structural components or inhibitors; specifically, Zn, Cu, Se and Mo act as essential cofactors for numerous enzymes, whereas Hg and As disrupt enzyme function and contribute to toxicity. Additionally, K, P, and S play vital roles in energy metabolism (such as ATP production), protein synthesis and cellular functions.

### 3.5 Elemental profiling in pancreatic tissue by ICP–MS

**Figure 5:**
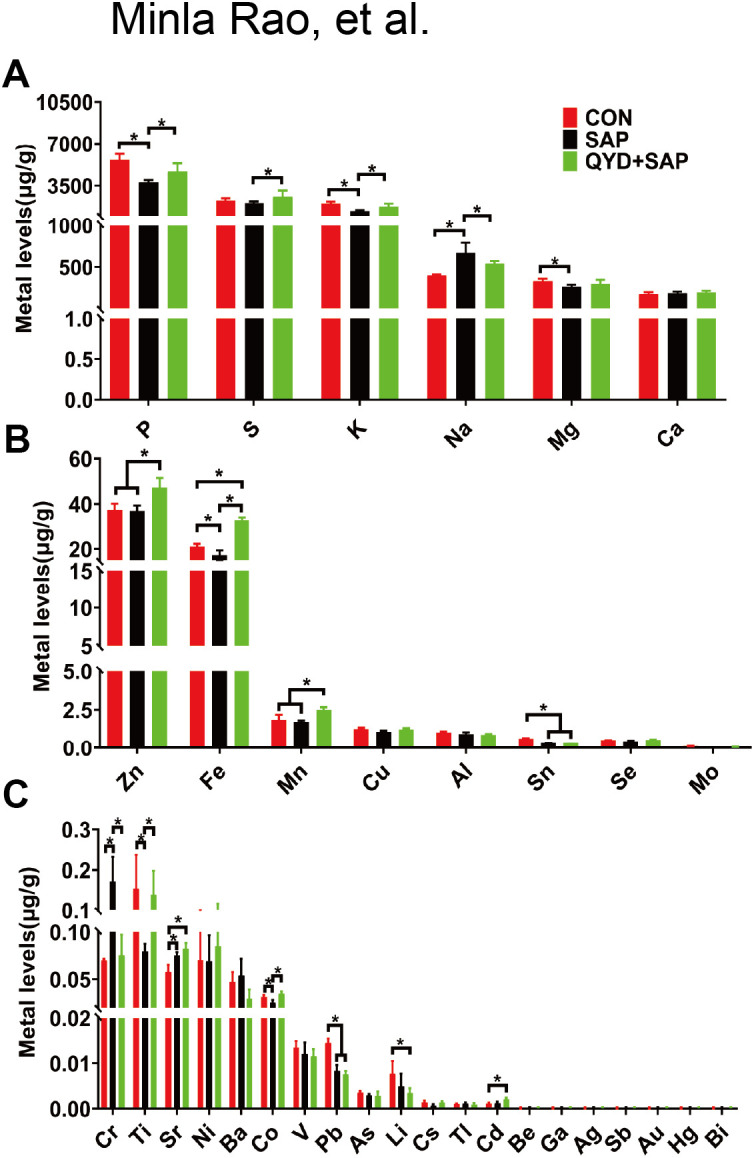
Elemental profiling in pancreatic tissue by ICP–MS. **(A)** Comparative analyses of ion concentrations in pancreatic tissues were conducted at high levels. **(B)** Comparative analyses of ion concentrations in pancreatic tissues were conducted at medium levels. **(C)** Comparative analyses of ion concentrations in pancreatic tissues were conducted at low levels. Data were expressed as mean ± *SD*; n=3 independent biological replicates; Statistical significance was determined by one-way ANOVA followed by Tukey’s *post hoc* test. (*P < 0.05).

To obtain a more systematic understanding of the distribution characteristics of ions in the body, we conducted a similar analysis of 34 elements in mouse pancreatic tissue using ICP–MS. In contrast to the findings in mouse serum, the elements that exhibited significant differences among the three groups were P, S, K, Na, Mg, Zn, Fe, Mn, and Sn, as illustrated in **Figures 5A–C**. The first five elements were present in greater quantities, whereas the last four appeared at lower concentrations. Our investigation revealed that, within both mouse serum and tissues, Zn is notably the most variable ionic element across the three groups, along with essential elements such as P, S, K, Na, and Mg.

### 3.6 Chemical speciation of metal ions in pancreatic tissue by XPS and AAS

**Figure 6:**
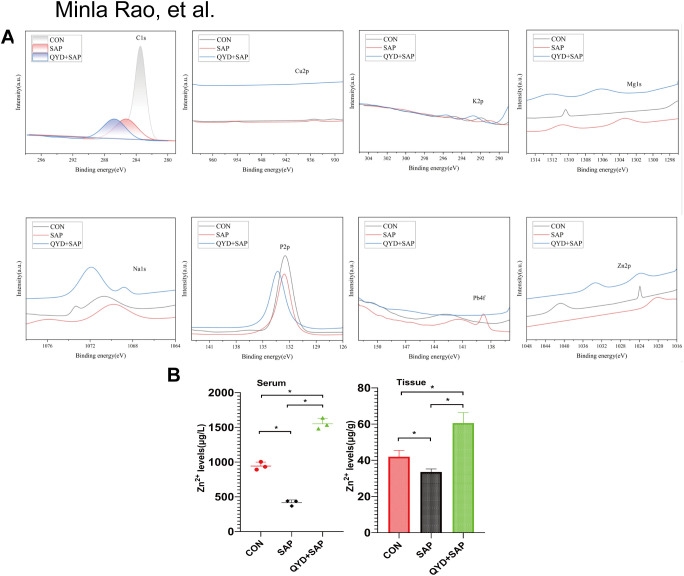
Chemical speciation of metal ions in pancreatic tissue by XPS and AAS. **(A)**XPS analysis of eight metal ions, as Cu, K, Mg, Na, P, Pb, Zn, in three groups. **(B)** Atomic absorption spectroscopy was employed to determine the concentration of zinc ions in serum and pancreatic tissue from mice across three groups. Data were as mean ± *SD*; n=3 independent biological replicates; Statistical significance was determined by Unpaired two-tailed Student’s *t*-test. (*P < 0.05).

To investigate the specific alterations in key metal ions, we employed XPS to analyse the variation trends of seven distinct metal ions (Cu, K, Mg, Na, P, Zn) across the three experimental groups. Our findings indicated that the Cu signal was weak and difficult to determine, but it was initially determined that the Cu in the SAP had a +2 valence. The binding energy of the K element was stronger in the QYD+SAP group, preliminarily indicating that QYD+SAP had a stronger capture ability for K^+^. The valence state of the K element in the three samples did not change. The Mg signal was significantly enhanced in QYD+SAP, with an increase in binding energy, indicating that Mg²^+^ in QYD may coordinate with the ligand. The Mg in the three samples most likely existed in the form of Mg²^+^. Regarding Na, the slight increase in binding energy of QYD+SAP indicated that Na^+^ was experiencing stronger coordination, but the change was minor, suggesting that there was likely no significant alteration in the valence state or species. Regarding P, the main peaks of CON and SAP essentially overlapped at approximately 133.2 eV, indicating that P was likely present in the form of phosphate. The slight increase in the binding energy of SAP suggested that the environment of SAP was more polar. The main peak of QYD+SAP shifted significantly towards a higher binding energy (approximately 134.0 eV), suggesting that it was more likely to coordinate with metals. Regarding the Zn element, the binding energies of the metallic and oxidized states of Zn 2p_3_/_2_ were very close, differing by only approximately 0.3 eV, which could easily lead to confusion and could not be used as a sole criterion for judgement. The binding energy of QYD+ SAP was greater than that of SAP, indicating that Zn²^+^ was likely undergoing coordination. The relatively broader peaks of QYD and SAP might also reflect the coexistence of multiple Zn ligand environments (**Figure 6A**).

To further investigate the elements with the most marked changes by XPS analysis, we collected serum and tissue homogenate supernatants from three groups of mice to determine Zn ion concentrations by AAS (**Figure 6B**). The results indicated that following QYD treatment, Zn ion concentrations were significantly elevated in both serum and tissue samples, suggesting the presence of multiple zinc ion ligands.

### 3.7 Identification of DEPs in pancreatic tissue

**Figure 7:**
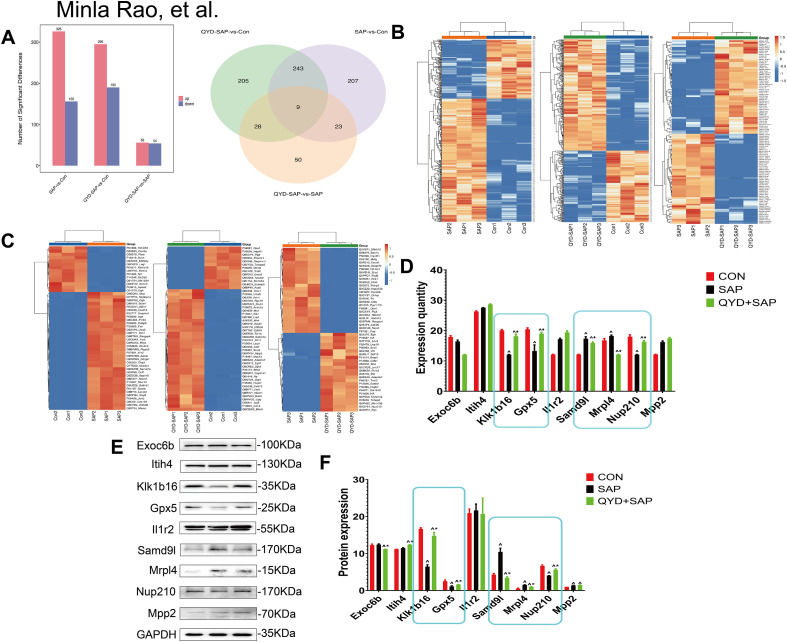
Identification of DEPs in pancreatic tissue. **(A)** Histogram of DEPs for SAP vs CON, QYD+SAP vs CON and QYD+SAP vs SAP: the x-axis represented comparison groups, and the y-axis represented the number of DEPs. Venn diagram of DEPs for SAP vs CON, QYD+SAP vs CON and QYD+SAP vs SAP. **(B, C)** Analysing nine differential protein expression with heatmaps. **(D)** Five proteins exhibited significant differences in expression levels across CON and SAP and QYD+SAP. n=3 biologically independent samples per group. **(E, F)** WB analysis results and quantitative graphs of nine proteins. Data were as mean ± *SD*; Statistical significance was determined by Unpaired two-tailed Student’s *t*-test. (^, vs CON, *P* < 0.05; ^*, vs SAP, *P* < 0.05).

In this study, compared with those in the CON group, 326 proteins in the SAP group were upregulated, and 156 proteins were downregulated. Compared with those in the CON group, 295 proteins in the SAP+QYD group were upregulated, and 190 proteins were downregulated. Compared with those in the SAP group, 56 proteins in the SAP+QYD group were upregulated, and 54 proteins were downregulated (**Figure 7A**). In both comparisons, the numbers of upregulated proteins were greater than those of downregulated proteins. As shown in the Venn diagram, 9 DEPs were found simultaneously in the three comparison groups (**Figure 7A**). Next, we examined the expression levels of the DEPs across the three groups (**Figure 7B**). A heatmap was then constructed to display the names of the top 20 differentially expressed proteins (**Figure 7C**).

To visually observe the expression levels of these nine core common proteins, we reproduced their expression levels in the proteomics data using a histogram. We observed that the expression levels of five proteins significantly differed across the three subgroups: Klk1b16, Gpx5, Samd9l, Mrp14, and Nup210 (**Figure 7D**). Additionally, we validated the differences in the expression of proteins across the three groups in the SAP mouse model by WB (**Figures 7E, F**). The WB results were consistent with the expression trends observed for the DEPs from the proteomics analysis, although the expression levels differed. Specifically, After QYD treatment, Klk1b16 and Gpx5 and Nup210 showed a significant upregulation. The five proteins whose expression levels significantly differed remained the same as those mentioned above. This consistency indicated that the bioinformatics analysis of the proteomics data did not exhibit significant bias.

### 3.8 GO and KEGG enrichment analyses of DEPs

**Figure 8:**
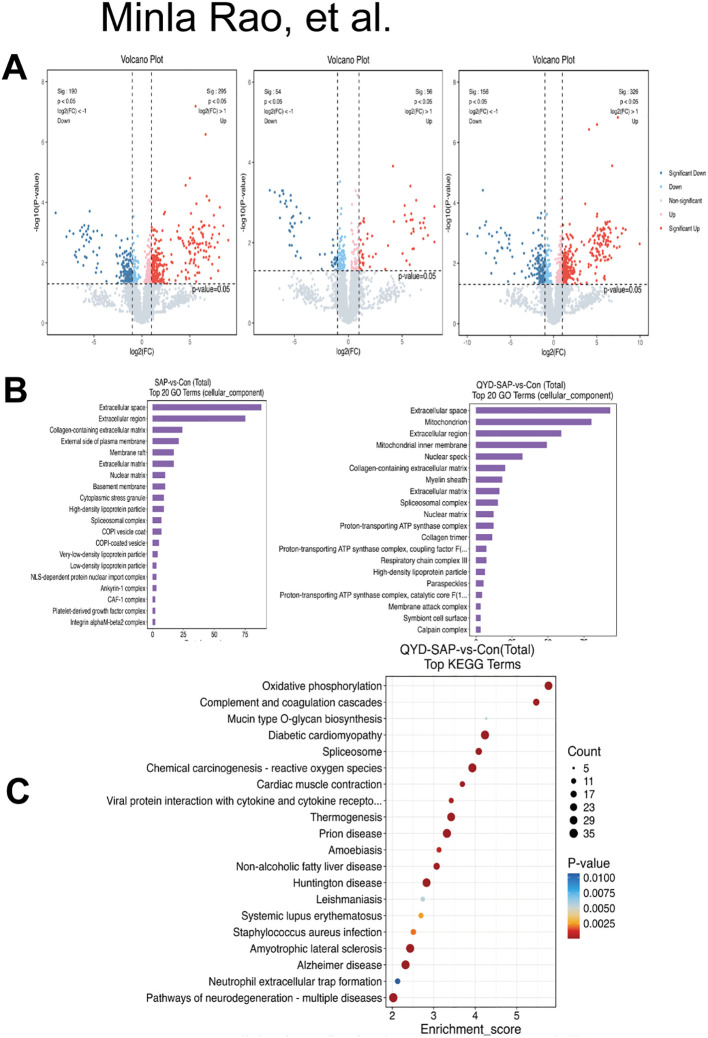
GO and KEGG enrichment analyses of DEPs. **(A)** Volcano plot displaying differentially expressed proteins in QYD+SAP vs CON and QYD+SAP vs SAP, SAP vs CON comparison. Proteins with |fold change| > 1.0 and FDR < 0.05 are highlighted in red (upregulated) and blue (downregulated). **(B)** GO pathway analysis results of DEPs for SAP vs CON, and QYD+SAP vs CON. **(C)** KEGG pathway analysis results for QYD+SAP vs CON.

To further illustrate the DEPs, volcano plots were constructed. The results are shown in **Figure 8A**; to understand the functions of the identified DEPs, we categorized them using GO enrichment analysis, which revealed three main categories: biological processes, cellular components, and molecular functions (**Figure 8B**). We then illustrated significantly enriched KEGG pathways (*P* < 0.05) using a bubble plot. Common enriched important KEGG pathways between QYD+SAP vs. CON included “oxidative phosphorylation,” “complement and coagulation cascades,” and “diabetic cardiomyopathy” (**Figure 8C**), of which “oxidative phosphorylation” was the most abundant.

### 3.9 PPI network analysis

**Figure 9:**
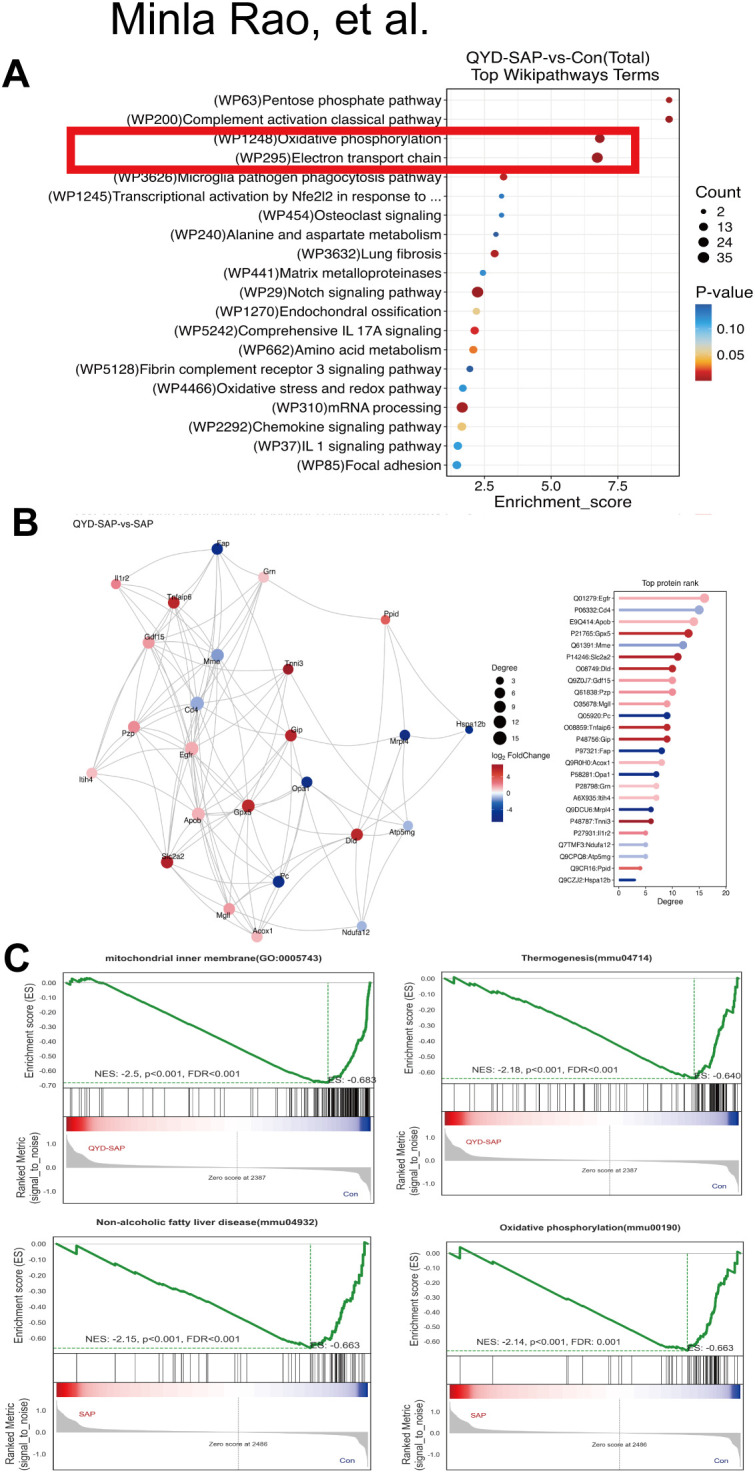
PPI network analysis. **(A)**Top Wiki pathways for QYD+SAP vs CON. **(B)** PPI network for QYD+SAP vs SAP. **(C)** biological processes for QYD+SAP vs CON and SAP vs CON.

A top Wiki pathways term diagram was constructed to illustrate the pathways enriched in the QYD+SAP and CON groups. Based on the results of the GO and KEGG analyses, the most distinctive features of these pathways were oxidative phosphorylation and the electron transport chain (**Figure 9A**).

Next, we analysed the protein interactions between QYD + SAP and CON using a PPI interaction network diagram. Among these, 25 proteins were relatively closely related (**Figure 9B**). We subsequently employed GO analysis to explore the biological processes associated with QYD+SAP and CON, specifically those pertaining to cellular energy metabolism. The results revealed that the energy flow process between QYD+SAP and CON involved the mitochondrial inner membrane and thermogenesis and that the energy flow process between SAP and CON involved oxidative phosphorylation and nonalcoholic fatty liver disease.

### 3.10 Correlation analysis between the ion spectrum and DEPs

**Figure 10:**
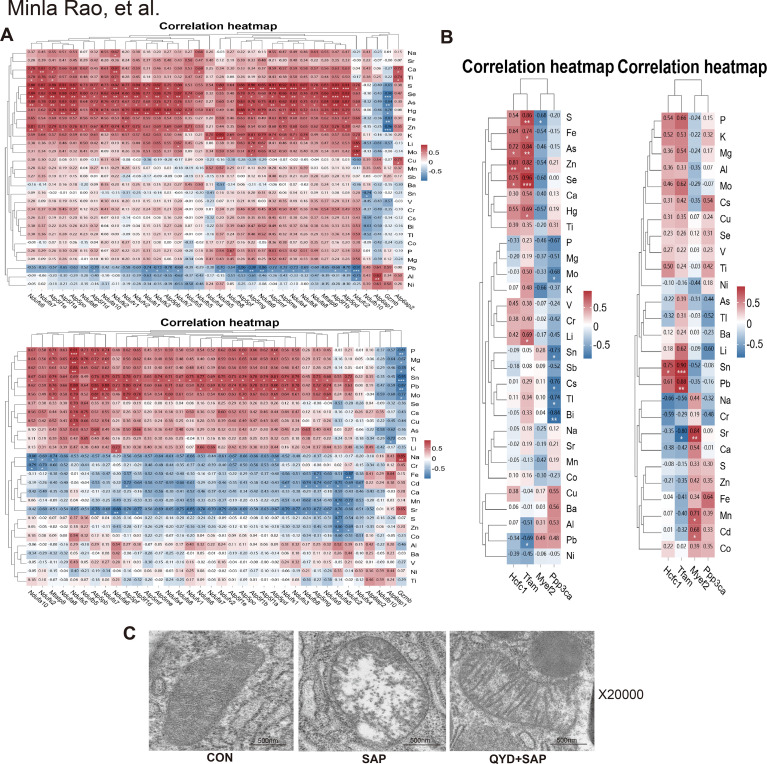
Correlation analysis between the ion spectrum and DEPs. **(A, B)** Correlation analysis between ion spectra and DEP involved in oxidative phosphorylation and mitochondrial energy metabolism. **(C)** Typical morphology of pancreatic tissue mitochondria under electron microscopy in CON group, SAP group, and QYD+SAP group (X20000). Scale bars: 500 nm.

To explore these data in more depth, integrated correlation analyses were conducted between the ionomic profile and DEPs involved in oxidative phosphorylation and mitochondrial energy metabolism (**Figures 10A, B**). The correlation heatmap revealed distinct clusters of DEPs associated with specific trace elements. Notably, Zn was positively correlated with multiple Nduf family proteins, suggesting that its role as a cofactor for antioxidant enzymes such as superoxide dismutase might extend to modulating respiratory chain activity through the upregulation of complex I subunits. Conversely, Cu was negatively correlated with Atp5f1b, indicating that excess Cu could suppress ATP synthase activity, which was consistent with the mitochondrial dysfunction observed in pancreatitis. Fe was positively correlated with Ndufv2, reflecting its essential function in maintaining iron–sulfur clusters for complex I integrity. Mn also correlated strongly with Ndufs4, potentially linking Mn-dependent antioxidant defence (via MnSOD) to respiratory chain stability.

Further analysis revealed mitochondrial transcription factor A (Tfam) as a central hub, with significant positive correlations with plasma Zn and tissue Fe. With respect to SAP, the levels of Zn, Fe, and Tfam were all decreased but were restored following QYD treatment, suggesting that Zn might directly support Tfam expression while Fe sustained mitochondrial bioenergetics. The levels of magnesium (Mg) and Mn also tended to increase with Tfam, likely through their roles in energy metabolism and oxidative defence. Se was positively linked to Tfam via antioxidant pathways. In contrast, Cu correlated negatively with Tfam, suggesting the inhibition of mitochondrial biogenesis. Additionally, Mg correlated positively with the signalling enzyme Ppp3ca, whereas tissue Ca showed a negative association, potentially reflecting pathological calcium overload in SAP. These findings emphasize that maintaining the homeostasis of trace elements—particularly Zn, Fe, and Mg—is crucial for preserving mitochondrial function via Tfam regulation.

To validate these correlative findings, mitochondrial morphology in pancreatic tissue was examined using electron microscopy (**Figure 10C**). Compared with the control group, the SAP samples exhibited severe mitochondrial damage, including swelling, cristae disruption, membrane fragmentation, and mitophagy. Treatment with QYD markedly attenuated these abnormalities, restoring mitochondrial structure, cristae organization, and membrane integrity. Together, these results demonstrated that mitochondrial dysfunction is a key event in SAP and that QYD might exert therapeutic effects by modulating ion homeostasis to preserve mitochondrial integrity and function.

## 4 Discussion

**Figure 11:**
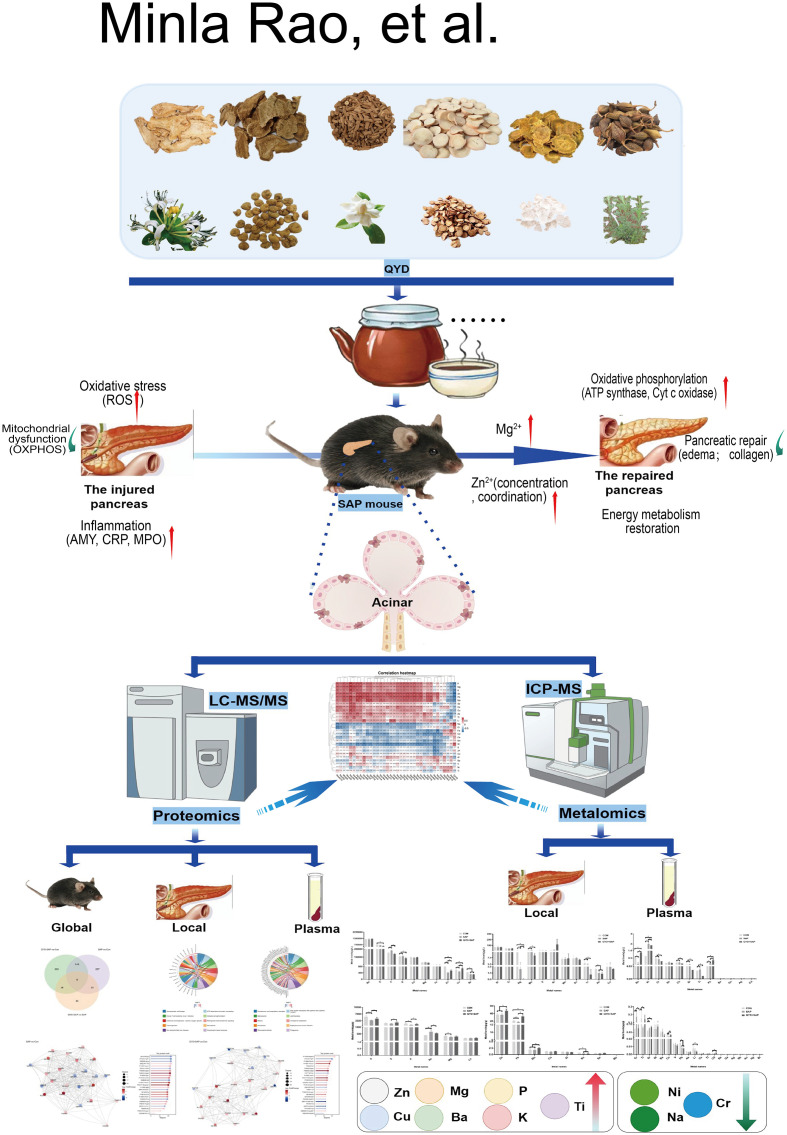
Schematic workflow of QYD treatment in mouse acute pancreatitis.

In this study (**Figure 11**), an inaugural integrative ionomic and proteomic analysis of a SAP mouse model with QYD was performed to explore the characteristics and potential links of the molecular mechanism of pancreatic repair. Our results demonstrated that QYD has multitarget therapeutic effects, including the alleviation of pancreatic inflammation, the restoration of metal ion homeostasis (particularly Zn), and the regulation of oxidative phosphorylation pathways. These results were highly consistent with recent literature, indicating that traditional Chinese medicine compound prescriptions could improve the prognosis of SAP through anti-inflammatory and metabolic regulation (20).

Qualitative compositional analysis validated the intricate makeup of QYD, aligning with its therapeutic profile characterized by multiple components and multiple targets. QYD had more multitarget effects than single-component drugs (such as pure zinc supplements) when dealing with complex systemic diseases such as SAP. The histological evidence of acute collagen deposition observed in the SAP group might reflect an early repair response after pancreatic injury, which also confirmed the success of the model construction (21).

The administration of QYD significantly attenuated the levels of inflammatory biomarkers (including CRP, MPO, and MCP-1) in both serum and tissue samples while concurrently reducing parenchymal infiltration of inflammatory cells such as macrophages. Furthermore, QYD treatment modulated the levels and valence states of trace metal ions, including Zn and Cu. Proteomic profiling revealed that QYD substantially altered the expression patterns of proteins associated with oxidative phosphorylation and mitochondrial function. Notably, strong correlations were observed between the levels of these metal ions and the expression of oxidative phosphorylation-related and mitochondrial proteins. These correlations were subsequently validated through assessment of mitochondrial morphology. These findings were consistent with the immunomodulatory potential of traditional Chinese medicine (22), indicating that our study provided molecular-level evidence for the therapeutic effect of QYD.

Notably, this study was the first to conduct such a large-scale analysis of ion change patterns in the SAP field, innovatively achieving dual-source comparisons between tissue and blood. Blood and tissue are measured simultaneously because in SAP, changes in blood ions often reflect overall stress and renal excretion status, whereas tissue ions reflect the local microenvironment. If blood zinc and tissue zinc increase synchronously, it indicates that QYD has a systemic regulatory effect on mineral homeostasis; if tissue zinc recovers faster than blood zinc does, it suggests that QYD promotes the “directed transport” of zinc to damaged organs. This dual-source comparison demonstrated that QYD not only had local anti-inflammatory effects but also exerted its effects through systematic network regulation, which was in line with the characteristics of the holistic theory of traditional Chinese medicine formulas. We also delved into the changes in ion coordination morphology and found that the regulatory changes in zinc ions were among the core findings of this study.

Zn deficiency exacerbates pancreatitis (23). This study revealed substantial changes in the coordination state of Zn^2+^ through XPS, and AAS further quantitatively confirmed the effective replenishment of the zinc pool by QYD. QYD effectively blocked the oxidative stress cascade in SAP. This might not involve a simple “zinc supplementation” but rather “precision-guided zinc rebalancing”.

Proteomic analysis revealed that oxidative phosphorylation was the most significantly enriched pathway regulated by QYD. The severity of SAP depends on whether acinar cells choose the “necrosis” or “apoptosis” pathway. Necrosis is disintegration, whereas apoptosis is programmed death. The key to deciding this choice is the ATP level (24). Oxidative phosphorylation is the main source of ATP (25). During SAP, oxidative phosphorylation paralysis leads to ATP depletion (26), forcing cells towards necrosis and the release of large amounts of DAMPs to exacerbate inflammation. Our research revealed that QYD enhanced intracellular ATP reserves by upregulating the expression of OXPHOS-related proteins such as ATP5A1. These findings indicated that QYD might shift the death mode of pancreatic cells from “proinflammatory necrosis” to “anti-inflammatory apoptosis” or directly rescue cells on the brink of death through energy repair. This explains why QYD could alleviate macroscopic inflammation.

By summarizing the specific DEPs after QYD treatment, we speculated that QYD might reverse the degradation of these key respiratory chain complex subunits, repair damaged mitochondrial transmembrane potentials, restore the energy supply to acinar cells, and prevent necrotic apoptosis. An integrated analysis of ionomic and proteomic data also revealed significant correlations between ion levels (Fe, Mg and Mn) and the mitochondrial transcription factor A (Tfam) of mitochondrial proteins. Tfam is not only a transcription factor for mitochondrial DNA (27) but also a protein that contains a high proportion of basic amino acids. Its stability and binding affinity to DNA are often regulated by microenvironmental ions. Moreover, many transcription factors contain zinc finger structures (28). Therefore, we speculated that the restoration of zinc levels by QYD might increase Tfam expression by activating the upstream PGC-1α signalling pathway (29). In this study, Tfam expression was significantly reduced in the SAP group, and although the expression in the QYD group did not fully recover, this finding was consistent with the trend of changes in ion levels, suggesting that ions might participate in the pathological process of pancreatitis by affecting the Tfam-mediated mitochondrial biogenesis mentioned above.

QYD not only reduces damage through traditional anti-inflammatory pathways but also, more importantly, activates Tfam-driven mitochondrial repair programs by reshaping ion homeostasis, which is the main role of zinc. This discovery challenged the traditional belief that traditional Chinese medicine could only suppress inflammation and revealed its deep potential in promoting the metabolic recovery of parenchymal organs.

Despite the above findings, this study has several limitations. First, although the sample size was sufficient to detect major differences, it might not be sufficient to capture subtle changes. Second, the findings in mice need to be validated in human samples. Third, proteomic analysis represents only a single time point; longitudinal studies could help reveal dynamic changes in disease progression and recovery processes. Fourth, although we detected a correlation between ion levels and DEPs, its causal relationship still needs to be functionally validated through more rigorous ion (for example, zinc) supplementation or chelation experiments. Fifth, there were limitations to the correlation analysis, but emphasizing this strong correlation (r=0.96) provides a highly credible hypothesis for future targeted therapy research. Future research should adopt a multiomics approach, including metabolomics and transcriptomics, to validate and expand these findings and explore the potential of ion supplementation in the treatment of SAP.

## 5 Conclusion

In summary, the results of the integrated study of ionomics and proteomics confirmed that QYD might improve mitochondrial function by reshaping the homeostasis of key metal ions (Zn, Cu, Fe, Ca, Mg, Mn, and Se; especially Zn) and targeting the repair of mitochondrial oxidative phosphorylation pathways, thereby exerting a protective effect on SAP. These findings provide not only high-resolution characteristics of ionomics and proteomics and molecular evidence for the clinical application of QYD but also a new perspective on the “ion metabolism–mitochondrial pathway and function” interaction in SAP pathogenesis.

## Data Availability

The original contributions presented in the study are included in the article/**Supplementary Material**. Further inquiries can be directed to the corresponding author/s.
