# Supplementary material for: Ionomics and proteomics analysis of the pancreatic repair in a murine severe acute pancreatitis model treated with Qingyi decoction
Source: Front Immunol. 2026 Apr 22;17:1797100. doi: 10.3389/fimmu.2026.1797100 (PMC13143770; doi:10.3389/fimmu.2026.1797100)
Supplement: Supplementary file 3 [file DataSheet3.zip › Supplementary Material 4:A certificate of editing/editing certificate.pdf]

Editing Certificate

This document certifies that the manuscript

Ionomics and proteomics analysis of the pancreatic repair in a murine severe acute pancreatitis model treated with Qingyi decoction

prepared by the authors

Minla Rao, Suzhen Zhang, Wenbin Lai, Di Zou, Zhihuang Wu, Zhenggang Yin, Wendie Yu,Weihua Zheng, Huimin Li, Yong Zhou, Wenjin Fu, Shayan Chen

was edited for proper English language, grammar, punctuation, spelling, and overall style by one or more of the highly qualified English speaking editors at AJE.

This certificate was issued on **March 16, 2026** and may be verified on the AJE website using the verification code **9BDF-0AC6-1830-EC07-343B** .

Neither the research content nor the authors' intentions were altered in any way during the editing process. Documents receiving this certification should be English-ready for publication; however, the author has the ability to accept or reject our suggestions and changes. To verify the final AJE edited version, please visit our verification page at aje.com/certificate.
If you have any questions or concerns about this edited document, please contact AJE at support@aje.com.

AJE provides a range of editing, translation, and manuscript services for researchers and publishers around the world.
For more information about our company, services, and partner discounts, please visit aje.com.
